# The Roles of Cytokinins in Plants and Their Response to Environmental Stimuli

**DOI:** 10.3390/plants9091158

**Published:** 2020-09-08

**Authors:** R. J. Neil Emery, Anna Kisiala

**Affiliations:** Department of Biology, Trent University, Peterborough, ON K9J 7B8, Canada; annakisiala@trentu.ca

**Keywords:** cytokinins, abiotic stress, biotic stress, environmental cues, thermoperiodicity, cold tolerance, drought tolerance, bacterial pathogen, phytophagous insects

## Abstract

Cytokinins (CKs) are adenine-derived, small-molecule plant growth regulators that control aspects of almost all plant growth and development processes. Internally, CKs play significant roles in plant cell division, nutrient allocation, and photosynthetic performance, and they are also detection and signaling agents for plant responses to the environmental challenges. CK functions in plant metabolism include plant adaptations to various abiotic stresses as well as their regulatory role in plant interactions with biotic components of the environment. Interestingly, CK biosynthesis is not exclusive to plants. New genetic and chemical approaches have revealed that both beneficial (symbiotic microorganisms) and detrimental (pathogenic bacteria, fungi, or insects) non-plant biota can secrete these phytohormones to purposefully modify plant metabolism. Therefore, while many open questions remain about how CKs are actively utilized by plants and plant-interacting organisms, CK roles should be seen more broadly, as signaling molecules for which effects range from within cells to as far as interkingdom relationships. The papers in this Special Issue highlight several aspects of CK biosynthesis, metabolism, and functions within plants and among plant-associated organisms, typifying the extensive range of roles played by these signaling molecules. The collection of papers represents new examples for CK researchers to consider advancing the growing range of topics related to how CKs mediate responses to many kinds of environmental stimuli and stresses.

As one of the classic groups of phytohormones, CKs have long been known for their potent promotion of plant growth. In fact, the original definition of a CK was for a compound which promoted cell division [1]. Over the decades since their early discovery in coconut milk, herring sperm and corn kernels, the cytokinins have gained a reputation for being involved in almost all growth processes from cellular metabolism to whole plant physiology, and even interactions with other organisms [2,3]. Though many uncertainties still exist around how CKs exert their effects, one thing that has become clear is that CKs are critical liaison elements between environmental signals and challenges and the orchestration of appropriate growth responses. With that in mind, this Special Issue called for works that advance our knowledge of the multilayered mechanisms of CK contribution both within plants and those involved in plant interactions with biotic and abiotic challenges faced during the plant life cycle. Topics ranged from proximal internal cues; to the distal, abiotic impositions of cold and drought; and, finally, to biotic interactions with pathogenic fungi and insect invaders. 

Flowering cues have been studied through the decades because of implications for the cut-flower industry and crop yield management. Many complex cues may exist, but select plant species somehow manage to sense and respond to the alternations of periods of heating and cooling. Thermoperiodic induction of reproductive tissue from apical meristems was studied by Tarkowska et al. [4] in this issue. The authors were following up on preliminary work that indicated CK levels increase in apices of winter rapeseed, which corresponded to transition times to reproduction during vernalization. To that end, they grafted vegetative apices of winter rapeseed onto reproductive winter and spring genotypes. Mass spectrometric analysis and microscopic observations indicated there were increases in specific forms of CKs during the early stages of apex floral induction. As development progressed, all forms of detected CKs proceeded to peak and decline, although floral apices always had higher CK levels than vegetative apices. The authors discussed those results and the implications for floral transitions in that thermoperiodic system. 

Thermoperiodism is an informative, yet non-stressful environmental cue that can ensure plant development progresses at the appropriate time for plant success. However, more extreme fluctuations in temperature demand an altogether different type of response to ensure damage is mitigated and survival better assured. In terms of potentially damaging cold temperature, plants can employ an array of responses but one of the most efficient is by scavenging free radicals that are produced by increasing rigidity of membrane systems, often associated with photosynthesis. Acidri et al. [5] explored the role of CK in the nonenzymatic antioxidant system within coffee, a crop which growers are seeking to expand into regions not yet suitable for cultivation due to cold intolerance. The authors treated coffee plants with a foliar spray of Kinetin, a form of CK rarely found in plants, but which usually has a high level of CK activity. Kinetin treated plants showed encouraging reactions, when challenged with cold temperatures, consisting of increased radical scavenging capacity and favorable photosynthetic responses such as higher gas exchange and greater photochemical CO_2_ fixation rates. These results hold promise as a practical solution for coffee growers.

Beyond serving as useful cues for appropriately timed developmental responses, if taken to more extremes, abiotic environmental variables can become stressful and harmful to normal growth. Drought is one such abiotic stress that limits global crop production, especially in countries and regions where irrigation is not practical or even possible. In terms of hormone response and drought mitigation, abscisic acid (ABA), has received most of researchers’ attention because of its known involvement in water conservation processes [6]. However, recent work involving CK over-producing transgenics has proven that CKs can be potent mitigators against drought challenges. In their review of the role of CKs in drought stress, Nguyen et al. [7] refer to these recent successes but they stress that we, in fact, know very little about the specifics of CK metabolic and signaling genes in drought stress. Furthermore, there is a blind spot regarding what is known about crosstalk with other signaling pathways and other important drought hormones such as ABA. They propose a genetic framework for the study of CK signaling and crosstalk along with precise monitoring of physiological drought responses. They conclude with a call for more basic knowledge of CK hormonal biology that can be applied for selection of new regulatory controls for plant drought adaptation. These authors answered their own call, to some extent, in a subsequent contribution to this Special Issue with a report on the effects of heterologous expression of a CK response regulator and apparent improvements to drought resistance [8]. This paper delves into the CK signal transduction pathway by manipulating a soybean response regulator (RR34) which operates as a potential drought-responsive agent situated downstream of the CK two-component system (TCS) of detection and transduction. The soybean RR34 was expressed ectopically in Arabidopsis to determine any possible role in drought mitigation. Results were promising and showed that GmRR34 operated in an ABA-associated mode of action and thereby improved water retention, antioxidant enzyme action and the regulation of several drought-responsive genes. Overall, the transgenics showed that manipulation of CK response regulators is a viable way to improve plant performance in the face of drought. 

Another layer of challenges experienced by plants is through their interaction with some other non-plant, members of the phytosphere, and this is manifested as biotic stress. Plant invaders can be bacteria, protists, fungi, or insects. Of course, many other organisms are friendly or beneficial—so, how can a plant respond differently to friend or foe? In a fascinating comparison, Jameson [9] comments on the differing CK profiles between virulent and benign strains of *Rhodococcus fascians*. The former is harmful and causes deformations of the plant and they live endophytically. The latter lives epiphytically but does not, in any obvious way, misdirect plant growth. The deformities caused by the virulent endophytes are thought to be the result of CK release into the plant. Yet, the epiphytes also release CKs to the plant, so what is the difference? The author concludes that it is the specific release of newly discovered methylated versions of CKs (Me-CKs). These unique bacterial CKs supercharge sugar and amino acid transport and metabolism to create inappropriate nutrient sinks, and thus morphological abnormalities.

Can this lesson in CKs from a bacterial phytopathogen also be applied to insect–plant interactions? This was put to the test in an ambitious survey of 17 species of phytophagous insects, including gall- and non-gall-inducing species from all six orders of *Insecta* that contain species known to induce galls [10]. Very little was known about the prevalence of CK production or its release by insects and the authors wanted to see if gall-inducing insects use some sort of unique CK system that could explain the strange growths they caused on plants. It turns out that all the phytophagous species tested produced an array of CKs. It was concluded that any plant-feeding insect can, in some way, use CK release to create more nutritious feeding sites. This is regardless of whether the insect in question caused obvious phenotypes like gall deformations or green islands. Apart from this general conclusion, other interesting research leads were generated. For example, insects only appear to have the tRNA-degradation pathway for CK production and lack the de novo adenylate pathway that is only found in higher plants. Yet, there is an unexpected diversity of CK forms in the insects of this study. Clearly there is much to learn in this new vein of CK discovery. 

In summary, this Special Issue contains an impressive collection of research articles, reviews and commentaries that cover a wide range of topics about CK responses to environmental stimuli. It features diverse systems for the study of environmental signals for development, abiotic stress, and biotic threats from bacteria and insects. Results give compelling reasons for readers to connect CK responses with their own research about environmental stimuli whether they are already engaged in the world of CK hormone biology. 
